# A foreign body experience

**DOI:** 10.1002/rcr2.606

**Published:** 2020-06-23

**Authors:** David Ferreira, Yashneel Prasad, Hima Vedam, Melissa Baraket, Sayontonee Ghosh, Jonathan P. Williamson

**Affiliations:** ^1^ South Western Clinical School Liverpool Hospital, University of New South Wales Sydney NSW Australia; ^2^ Respiratory, Sleep and Environmental Health Research Group South Western Sydney Academic Unit Sydney NSW Australia; ^3^ MQ Health Respiratory and Sleep Macquarie University Hospital Sydney NSW Australia

**Keywords:** Clinical respiratory medicine, cough, radiology and other imaging

## Abstract

Foreign body inhalation (FBI) is an uncommon clinical entity in adults. Despite this, FBI is an under‐recognized, serious, and easily treatable condition. The authors report a case of a 31‐year‐old female asthmatic who presented with wheeze and cough not responding to therapy. Chest computed tomography and bronchoscopy confirmed a foreign body distal to the left upper lobe vestibule. The non‐organic material was removed with rigid bronchoscopy and cryoprobe with resolution of symptoms. FBI remains an important differential in those presenting with respiratory symptoms in the absence of other diagnoses.

## Graphical abstract

Foreign body inhalation (FBI) is an uncommon clinical entity in adults and is a potentially serious but treatable condition. The authors report a case of a 31‐year‐old female asthmatic who presented with FBI. A non‐organic object was removed with rigid bronchoscopy and a cryoprobe with resolution of symptoms.

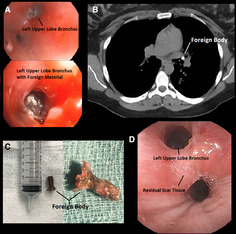

## Introduction

While inhaled foreign bodies are seen regularly in the paediatric population, they are rare in adults. Foreign body inhalation (FBI) can be defined based on anatomical location (proximal or distal bronchial tree) as well as the inhaled material (organic or non‐organic). Proximal obstruction is common in children due to airway anatomy while distal deposition of foreign bodies is more likely in adults [1]. High index of suspicion is required for diagnosis and, due to non‐specific symptoms, there is often delay in definitive management.

The authors report a case of a non‐organic, distal bronchial tree FBI in an adult patient. Clinical presentation, diagnosis, and management options are then discussed for this rare, yet potentially life‐threatening, occurrence.

## Case Report

**Figure 1:**
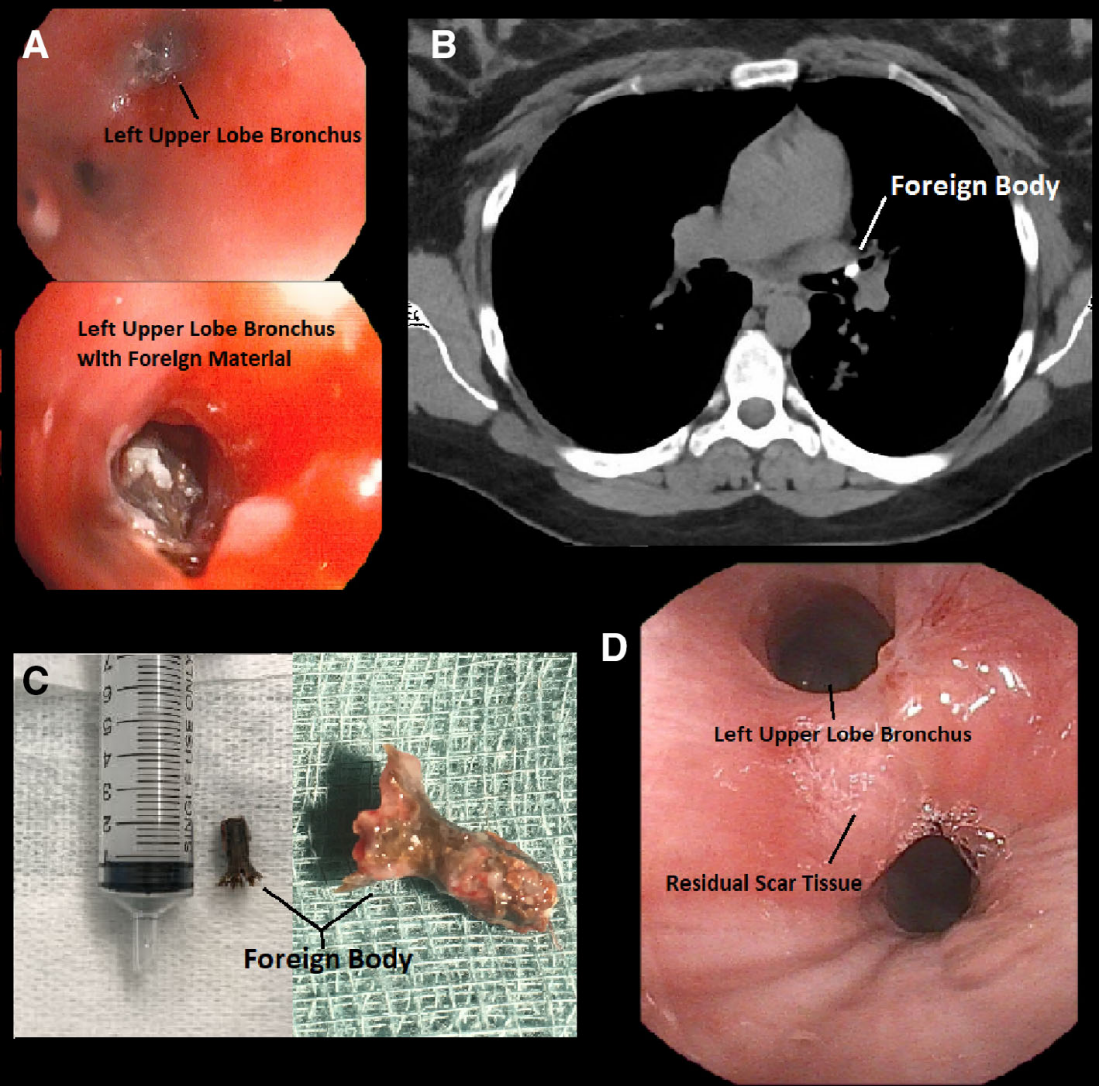
(A) Foreign body in the left upper lobe bronchus. (B) Foreign body identified on chest computed tomography. (C) Non‐organic foreign body. (D) Follow‐up bronchoscopy demonstrating patent left upper lobe bronchus.

A 31‐year‐old female presented with a three‐week history of expiratory wheeze and cough. Background history was significant for pre‐eclampsia and gestational diabetes with no prior respiratory issues. Her symptoms persisted despite bronchodilators, oral corticosteroids, and antibiotics provided by her primary care physician. Further history revealed the symptoms were preceded by an episode of vomiting and aspiration while eating cereal. Chest auscultation demonstrated a monophonic wheeze worse on of the left. Flexible bronchoscopy performed under sedation revealed marked narrowing and mucosal inflammation in the left upper lobe with granulation tissue and foreign material distal to the upper lobe vestibule (Fig. 1A). The left lower lobe orifice was also circumferentially narrowed. Attempts to remove the foreign body with flexible biopsy forceps were abandoned due to coughing, localized bleeding, and transient hypoxaemia. Non‐contrast chest computed tomography following the bronchoscopy showed a hyperdense occlusive object in the left upper lobe bronchus (Fig. 1B). A subsequent rigid bronchoscopy was performed. Obstructing granulation tissue was debulked with argon plasma coagulation diathermy to improve the view of the foreign body which was then removed using a 1.9‐mm cryoprobe following a 4‐sec freeze time. The foreign body was identified as non‐organic plastic material (Fig. 1C). Bronchoscopy performed three weeks after removal demonstrated reduced airway inflammation with improving bronchial diameters and minor residual scar tissue (Fig. 1D). The patient has since made a full symptomatic recovery. Attempts to identify the origin of the foreign body have been unsuccessful.

## Discussion

There is a broad range of clinical manifestations with FBI, ranging from asphyxia with cardiac arrest to chronic cough. Those with proximal, laryngotracheal foreign bodies commonly present with breathlessness, cough, and stridor. Those with distal, bronchial foreign bodies generally have symptoms of wheeze, shortness of breath, and cough. Rarer manifestations include recurrent pulmonary infections, haemoptysis, and even isolated fever [2]. In patients with respiratory symptoms without another diagnosis, careful screening history for FBI is warranted. Due to physician error, there is often a delay in diagnosis which can range from days to months, highlighting the need for a high index of suspicion. Delays in diagnosis and management increase long‐term complications: pneumonia, emphysema, and bronchiectasis.

Chest radiography is notoriously insensitive for FBI and further investigations should be pursued if there is clinical suspicion [3]. Chest computed tomography is helpful; however, it is associated with both false‐negative and false‐positive results [4]. FBI is generally confirmed by flexible bronchoscopy with direct visualization [1].

Flexible bronchoscopy remains the standard of management with a success rate of approximately 80–90% [5]. Rigid bronchoscopy is a reasonable treatment option following failed flexible bronchoscopy, and is associated with high success rates. When bronchoscopic intervention fails, thoracotomy or lobectomy may occasionally be required [4]. Consideration should be made for follow‐up chest imaging and repeat bronchoscopy to exclude residual foreign body and malignancy.

Although uncommon, the recognition and timely management of FBI improves patient symptoms and reduces long‐term complications. Flexible bronchoscopy is the cornerstone of diagnosis and management. FBI should be considered in those with respiratory symptoms without an alternative diagnosis or refractory to treatment.

### Disclosure Statement

Appropriate written informed consent was obtained for publication of this case report and accompanying images.
